# Insulin in Type 1 and Type 2 Diabetes—Should the Dose of Insulin Before a Meal be Based on Glycemia or Meal Content?

**DOI:** 10.3390/nu11030607

**Published:** 2019-03-13

**Authors:** Janusz Krzymien, Piotr Ladyzynski

**Affiliations:** Nalecz Institute of Biocybernetics and Biomedical Engineering of the Polish Academy of Sciences, 4 Trojdena Street, 02-109 Warsaw, Poland; janusz.krzymien@hotmail.com

**Keywords:** carbohydrate counting, protein and fat counting, insulin dosage, glucose monitoring, diabetes mellitus, type 1 diabetes, type 2 diabetes

## Abstract

The aim of this review was to investigate existing guidelines and scientific evidence on determining insulin dosage in people with type 1 and type 2 diabetes, and in particular to check whether the prandial insulin dose should be calculated based on glycemia or the meal composition, including the carbohydrates, protein and fat content in a meal. By exploring the effect of the meal composition on postprandial glycemia we demonstrated that several factors may influence the increase in glycemia after the meal, which creates significant practical difficulties in determining the appropriate prandial insulin dose. Then we reviewed effects of the existing insulin therapy regimens on glycemic control. We demonstrated that in most existing algorithms aimed at calculating prandial insulin doses in type 1 diabetes only carbohydrates are counted, whereas in type 2 diabetes the meal content is often not taken into consideration. We conclude that prandial insulin doses in treatment of people with diabetes should take into account the pre-meal glycemia as well as the size and composition of meals. However, there are still open questions regarding the optimal way to adjust a prandial insulin dose to a meal and the possible benefits for people with type 1 and type 2 diabetes if particular parameters of the meal are taken into account while calculating the prandial insulin dose. The answers to these questions may vary depending on the type of diabetes.

## 1. Introduction

In healthy people, fasting plasma glucose rarely reaches 5.5 mmol/L (100 mg/dL) and the highest values after meals do not exceed 7.8 mmol/L (140 mg/dL), and quickly return to the starting level [1]. Hyperglycemia defines diabetes, and glycemic control plays an important role in the treatment of diabetes. Type 1 and type 2 diabetes are the two main types of the disease, which affects more than 425 million people worldwide [2]. Consistent hyperglycemia can lead to serious micro- and macrovascular complications, which cause diseases affecting the heart and blood vessels, kidneys, eyes, nerves and teeth. In addition, people with diabetes also have a higher risk of developing infections. In almost all high-income countries, diabetes is a leading cause of cardiovascular disease, blindness, kidney failure, and lower limb amputation. The premature morbidity, mortality, reduced life expectancy, and financial and social costs of diabetes make it one of the most important public health conditions [3]. The goals of treatment for diabetes are to prevent or delay complications, decrease mortality and maintain quality of life. An adequate glycemic control is a way of achieving these goals. In the American Diabetes Association (ADA) recommendations [4] for the majority of adults with diabetes (excluding pregnant women), the glycated hemoglobin A1c (HbA1c) below 7.0% (53 mmol/mol), preprandial blood glucose concentration in the range from 4.4 to 7.2 mmol/L (from 80 to 130 mg/dL) and after meals—below 10.0 mmol/L (180 mg/dL) were accepted as target values. At the same time, the recommendations underline that more or less stringent targets may be taken into account. They should be individualized depending on the duration of diabetes, age, life expectancy, co-morbid medical conditions, cardiovascular complications or advanced microvascular complications, occurrence of the hypoglycemic unawareness, as well as the individual expectations of the person with diabetes. It should be noted that in recent years, organizations such as The National Institute for Health and Care Excellence (NICE) [5], Institute for Clinical Systems Improvement (ICSI) [6], American Association of Clinical Endocrinologists and American College of Endocrinology (AACE/ACE) [7,8], Scottish Intercollegiate Guidelines Network (SIGN) [9], and the U.S Veterans Affairs/Department of Defense (SIGN) [10] have indicated that the target values of HbA1c are differentiated depending on the individual characteristics of people with diabetes. In its recently published recommendations, the American College of Physicians proposed target values of HbA1c for most people with type 2 diabetes in the range of 7.0% to 8.0% (53 to 64 mmol/mol) [11]. In a commentary from Diabetes Care, Matthew C. Riddle and colleagues [12] indicate the necessity to evaluate established and newer therapeutic options based on many years of observation, which will optimize the individualization of both goals and methods of therapy. The therapeutic goals set by ADA seem balanced and reasonable, they are broadly in line with the guidelines of other scientific societies. The HbA1c concentration below 7.0% (53 mmol/mol) is the accepted target value for the majority of patients with diabetes, excluding pregnant women. The possibility of achieving a more stringent goal of treatment, i.e., HbA1c below 6.5% (48 mmol/mol) for a group of patients who can achieve this goal without an increased risk of hypoglycemia or other adverse effects of the treatment as well as taking into account less stringent values of HbA1c, e.g., below 8.0% (64 mmol/mol) for older people are good examples of individualized therapeutic goals. Recommendations of the Polish Diabetes Association include similar individualized therapeutic goals [13]. Therapy adjustments should be made to maximize the proportion of time that glycemia is within the optimal range, i.e., between 3.9 and 10.0 mmol/L (70 and 180 mg/dL) for most patients [14]. A study of Bode et al. in people with type 1 and type 2 diabetes with HbA1c of 7.5% (58 mmol/mol) showed that 29% of blood glucose values exceeded 10.0 mmol/L (180 mg/dL) [15]. The postprandial blood glucose testing and evaluation is particularly important if the HbA1c target is not achieved despite satisfactory pre-meal glycemia. The postprandial glucose measurements should be made 1–2 h after the beginning of the meal and using treatments aimed at reducing postprandial plasma glucose values to <10.0 mmol/L (180 mg/dL) may help to lower HbA1c [4].

What is the real effect of the diet on the postprandial glycemia? Can we assess the effect of carbohydrates, fat and protein, and meals with a high or low glycemic index (GI) on postprandial glycemia? Are there differences that are related to the type of diabetes? In this review, we have attempted to answer these questions, based on the existing guidelines and available scientific evidence, to see if the prandial insulin dose should be calculated based on glycemia or the size and composition of a meal including carbohydrates, protein and fat content in the meal. We investigated the effect of macronutrient content in a meal on postprandial glycemia and we identified factors that may make it difficult to determine the appropriate dose of insulin to compensate for food intake in people with diabetes. Then we reviewed effects of the existing insulin therapy regimens on glycemic control and we assessed existing algorithms aimed at calculating prandial insulin doses in people with type 1 and type 2 diabetes. Finally, we concluded the work and asked questions which are still waiting for answers.

## 2. Effect of Fat, Protein and Carbohydrates in a Meal on Postprandial Glycemia

In type 1 diabetes, fat reduces the early glucose response (the first 2–3 h after the meal) and delays the peak blood glucose due to the delayed gastric emptying. In type 1 diabetes, fat leads to late post meal (>3 h) hyperglycemia [16]. In children with type 1 diabetes, adding 35 g of fat to the meal can increase the blood glucose level by 2.3 mmol/L (41 mg/dL) [17]. Fat consumption increases the need for insulin and requires an individual increase of the insulin dose with caution, and the calculation of the necessary insulin dose should take into account the duration of insulin action [18]. While paying attention to prevent late after-meal hyperglycemia, one should remember the risk of early hypoglycemia immediately after the meal. Campbell et al. demonstrated in type 1 diabetic patients that increasing the mealtime insulin is not an efficient strategy alone because it may increase the risk of early postprandial hypoglycemia [19].

Total and saturated fat intake were associated with a higher risk of type 2 diabetes, but these associations were not independent of obesity [20]. Dietary fat and free fatty acids (FFA) are known to impair insulin sensitivity and enhance hepatic glucose production [21]. Limited results are available on the fat effects on gastric emptying and postprandial glycemia in people with type 2 diabetes. Gentilcore et al. demonstrated that ingestion of fat before a carbohydrate meal resulted in slower gastric emptying and attenuated postprandial rises in glucose, insulin, and glucose-dependent insulinotropic polypeptide but it stimulated glucagon-like peptide-1 (GLP-1) in type 2 diabetes [22].

Protein consumption affects the blood glucose concentration in the late postprandial period. In people with type 1 diabetes using intensive insulin therapy 75 g or more of protein alone significantly increases postprandial glycemia from 3 to 5 h in people with type 1 diabetes [23]. Moreover, protein has different effects when consumed with and without carbohydrates, e.g., 30 g of protein with carbohydrates will affect blood glucose [16,17].

In type 2 diabetes, protein when consumed without carbohydrates has a very small effect on the level of glucose in the blood. In people with type 2 diabetes, after ingesting 50 g of protein the glucose response to protein remains stable for 2 h and then begins to decline [24]. Protein does not result in an increase of the blood glucose concentration, and it results in only a modest increase in the rate of glucose disappearance [25].

Meals with a high GI cause a rapid increase in glycemia. An inadequate insulin dose that does not take into account the rapid absorption of carbohydrates after consuming foods with a high GI may lead to a rapid increase in the blood glucose concentration.

Foods with a low GI cause a smaller increase in the glucose level, reducing the peak of glycemia, but at the same time it was demonstrated that such foods increase the risk of hypoglycemia after the meal in people with type 1 diabetes if taken with inadequate doses of insulin [16]. Three studies in people with type 1 diabetes suggested that the risk of mild hypoglycemia is greater with low GI than with high GI foods when the usual carbohydrate-to-insulin ratio is used [26,27,28]. However, low GI diets can significantly improve metabolic control in less than optimally controlled people with type 1 and type 2 diabetes, as indicated by the results of a meta-analysis reported by Thomas and Elliott [29]. The twelve included studies in this meta-analysis involved a total of 612 participants. Three studies had participants with type 1 diabetes, 8 studies had participants with type 2 diabetes, and one study had participants with either type 1 or type 2 diabetes. Low GI diets lower HbA1c levels by 0.4% compared with comparison diets, i.e., high GI diet in 10 studies, measured carbohydrate exchange diet in one study, and a high-cereal fiber diet in another study. Nevertheless, it should be remembered that eating a large amount of high carbohydrate foods with a low GI can lead to a significant increase in glycemia. A similar effect is observed after eating foods with a low GI but with a high content of fructose or sucrose, e.g., fruit juices.

The following questions should be asked: what do we know about the effects of individual components of the diet on postprandial glycemia in people with diabetes and is this knowledge necessary for us to effectively control it?

In a study reported in 2004, the effect of two diets on a 24-h glycemia and insulinemia profiles was compared in people with untreated type 2 diabetes [30]. The control diet was developed in accordance with the recommendations of the American Heart Association and the US Department of Agriculture, and it consisted of 55% carbohydrates with emphasis on products containing starch, 15% protein and 30% fat, including 10% monounsaturated, 10% polyunsaturated and 10% saturated fatty acids. The test diet was designed to consist of 20% carbohydrates, 30% protein and 50% fat. The content of saturated fatty acids in this diet was ~10% of the total food energy. Mean blood glucose values determined after 5 weeks of the dietary treatment were significantly lower in patients using the test diet compared to those using the control diet, 7.0 mmol/L (126 mg/dL) vs. 10.5 mmol/L (190 mg/dL). The control diet caused a higher increase of the insulin concentration in the blood. Thus, the diet with the carbohydrate restriction consumed for 5 weeks dramatically reduced blood glucose levels in people with type 2 diabetes. However, it should be emphasized that the study was conducted in people with untreated diabetes. Studies on the effect of various diets on glycemic control in people with type 2 diabetes treated with insulin have not been conducted so far, and thus—are required. Low-carbohydrate diets (LCD) are recognized as “no side effects” diets in the newest consensus report of ADA and the European Association for the Study of Diabetes (EASD) [31]. However, in the same consensus report the systematic literature review and meta-analysis of Sainsbury et al. is cited, which indicates that LCDs (26% of total energy) in people with type 1 and type 2 diabetes produce substantial reductions in HbA1c at 3 months and 6 months, with diminishing effects at 12 and 24 months and that no benefit of moderate carbohydrate restriction (26%–45%) was observed in 25 randomized controlled trials involving 2415 participants that were included in the meta-analysis [32]. Moreover, Mizidi et al. demonstrated the unfavorable effect of LCDs on total and cause-specific mortality, based on both individual data and pooling previous cohort studies. These authors concluded that given the fact that LCDs may be unsafe, it would be currently preferable not to recommend such diets [33]. On the other hand, several cohort studies showed that high carbohydrate diets (HCD) might be associated with higher risk of total mortality and cardiovascular disease [34,35].

The composition of the diet isn’t the only factor that affects the blood glucose after a meal. In people with poorly controlled type 2 diabetes, reducing consumption of carbohydrates during breakfast and increasing it during lunch helps to improve glycemic control. By choosing one of meals with the highest carbohydrate content, while keeping the same daily intake of carbohydrates, a different therapeutic effect can be obtained [36].

The order in which foods containing various amounts of carbohydrates, protein and fat are consumed also affects the postprandial glucose level. Shukla et al. studied people with type 2 diabetes, who have been receiving the same meal consisting of carbohydrates, protein and vegetables for three days and consumed it in a random order: first carbohydrates, then after 10 min protein and vegetables or first protein and vegetables, then after 10 min carbohydrates or all components of the meal at the same time. It was shown that peaks of the glucose rise after the meal were more than 50% lower, with a simultaneously lower increase of insulinemia and higher secretion of GLP-1 when carbohydrates had been consumed as the last part of the meal in comparison with consumption of carbohydrates at the beginning of the meal [37]. The order of consuming food products affects also concentration of ghrelin—the “hunger hormone” which has orexigenic and adipogenic properties and is thought to play an important role in regulating food intake and body weight. Shukla et al. showed that the intake of carbohydrates at the beginning of the meal led to the restoration of postprandial ghrelin concentration in the postprandial period [38]. This effect was not found when the meal was started by serving protein and vegetables. Ghrelin secretion is suppressed immediately after a meal, the depth and duration of the suppression being proportional to the energy intake. Insulin may suppress circulating ghrelin independently of glucose [39]. Taking carbohydrates at the beginning of a meal shortens the period of suppression of ghrelin, which may result in shortening the period of feeling satiety, speeding up the next meal, and consequently leading to weight gain.

All these data indicate that a few factors may influence the increase in glycemia after the meal, which creates significant practical difficulties in determining the appropriate dose of insulin to compensate for food intake in people with diabetes.

## 3. Automatic Bolus Calculators in People with Type 1 Diabetes

People with type 1 diabetes treated with insulin pumps may use an automatic bolus calculator that allows them to determine the insulin dose based on the amount of carbohydrates consumed in the meal and an individual insulin sensitivity. The use of the bolus calculator in patients treated with multiple insulin injections resulted in a significant improvement of the metabolic control while the number of hypoglycemic episodes was slightly reduced [40]. There are some promising new applications of the emerging technologies in this field like the expert system using the automatic speech-to-text conversion, which is able to determine the caloricity, the content of carbohydrates, fat and protein in the meal in a fairly accurate way based on its voice description provided by the user. Such a system is an easy-to-use support tool in the type 1 diabetes treatment that makes it possible to improve the postprandial glycemic control [41,42]. This system can also be useful in people with type 2 diabetes to control the amount of food consumed or adjust insulin dosage. Other systems that attempt to calculate the meal content based on its digital image [43,44] or the monitoring of activities related to consumption of the meal, e.g., swallowing or chewing [45] are also under development and testing. Regardless of how the meal content is determined and entered, each automatic bolus calculator must implement an algorithm determining the insulin dose, which should be able to control postprandial glucose concentration. The review of such algorithms for people with type 1 diabetes can be found for example in a report of Krzymien et al. [46].

In people with type 1 diabetes, prandial insulin doses are individualized using parameters such as the insulin-to-carbohydrates ratio, and much less frequently, the circadian fluctuations of this parameter. However, in most algorithms the meal is characterized just by carbohydrate content despite the fact that new insights concerning the effect of dietary macronutrients on postprandial glycemia confirm that fat and protein content should be taken into consideration while calculating prandial insulin doses [16,47,48]. Pankowska and Blazik showed that the insulin bolus calculator with an algorithm accounting for carbohydrates and protein and/or fat in the meal could effectively suggest a normal or a square-wave bolus and indicate the timing of the square-wave bolus in the insulin pump users [49]. The same authors demonstrated in a 3-month open label randomized control study that the use of this system by educated children and adolescents with type 1 diabetes was safe and reduced 2-h postprandial blood glucose level and glucose variability [50].

Summing up, the improvement in the postprandial plasma glucose control in people with type 1 diabetes depends primarily on properly adjusting the insulin dose to the meal being consumed.

## 4. Inter-Subject Variability of a Response to Meals

The algorithms that have been implemented in automatic bolus calculators so far have not accounted for variability in the response to meals with identical carbohydrate content. In 2015, Zeevi et al. demonstrated on an 800-person cohort of individuals aged 18–70 not previously diagnosed with diabetes that people eating identical meals presented high variability in post-meal blood glucose responses. These authors showed that personalized diets created with the help of an accurate predictor of the blood glucose response integrating parameters such as dietary habits, physical activity, and gut microbiota might successfully lower postprandial blood glucose concentration and its long-term metabolic consequences [51]. In the same report authors developed a machine-learning algorithm accurately predicting personalized postprandial glycemic responses to real-life meals based on blood parameters, dietary habits, anthropometrics, physical activity, and the gut microbiota. These results indicate that it may be beneficial to incorporate such a personalized meal-dependent predictor of the postprandial glycemia into automatic insulin bolus calculators. However, we must emphasize that in the work of Zeevi et al., the study group consisted of a healthy population and it should be confirmed that the findings are equally valid in people with diabetes before they can be used to optimize the diabetes treatment. Recently, Rozendaal et al. demonstrated that the large variability in postprandial glycemic response dynamics to different types of food is inadequately predicted by existing glycemic measures such as the Glycemic Index, the Glycemic Load and the Glycemic Glucose Equivalents [52]. They quantitatively described the postprandial glycemic response dynamics using a physiology-based dynamic model. Although both these reports were based on data of people without diabetes, their conclusions admittedly should be applicable also to people with type 1 diabetes. The conclusions from the recent studies on variability of postprandial glucose response, if confirmed in people with diabetes, should result in more personalized algorithms for prandial insulin dose calculation in the future.

## 5. Insulin Therapy in People with Type 2 Diabetes

Insulin therapy in people with type 2 diabetes is significantly different from that used to treat people with type 1 diabetes. Typically, the treatment starts with the basal insulin and is directed to the fasting blood glucose control [53]. The fasting glucose is closely controlled by regulating hepatic glucose production with variable release of insulin into the portal vein, and with modulation of insulin action in the liver by glucagon and FFA [54,55]. In type 2 diabetes, the basal insulin secretion is impaired, and FFA and the fasting glucagon levels are high. An injection of long-acting insulin inhibits glucose production in the liver by acting directly on the liver and indirectly by reducing the release of FFA from adipose tissue [56,57]. In untreated type 2 diabetes, the HbA1c level depends primarily on fasting glycemia. The postprandial glucose concentration is less influential, especially in cases where the HbA1c level is greater than 8.0% (64 mmol/mol) [58]. In type 2 diabetes, most of the hypoglycemic agents used allow effective control of the fasting blood glucose. Metformin, sulfonylureas, thiazolidinediones and basal insulins (human NPH and insulin glargine, detemir, degludec) have little effect on postprandial hyperglycemia. Starting the basal insulin therapy is very simple. Normally, oral medications that have been used before initiation of the insulin therapy are given with insulin at a dose of 10 U or 0.1–0.2 U/kg of the body weight. The daily dose is then adjusted based on the fasting glycemia to obtain an individually determined fasting glucose target by increasing it in steps of 10% to 15% or 2 to 4 U once or twice a week. In the case of hypoglycemia, the dose is usually reduced by 4 U or 10% to 15% [59]. Yki-Järvinen et al. performed a study in patients with type 2 diabetes treated with one or two oral preparations with an average baseline HbA1c value of 9.5% (80 mmol/mol). After 36 weeks of active administration of insulin glargine and metformin, a significant improvement in metabolic control and a reduction in the fasting glucose was achieved. During the last 12 weeks the fasting plasma glucose averaged 5.8 mmol/L (104 mg/dL), and the mean HbA1c value was 7.14% (54 mmol/mol) at the end of the study period implying that half of the study group remained inadequately controlled (HbA1c > 7%), mainly due to hyperglycemia during the day [60]. The insulin treatment of people with type 2 diabetes requires normalization of both fasting and postprandial glycemia [61]. Failure to achieve HbA1c targets requires intensification of the therapy by choosing one of two therapeutic options, administering insulin mixtures twice a day, or administering fast-acting insulin before the largest meal.

For the treatment with insulin mixtures, the following principles established by ADA should be followed in the majority of people with type 2 diabetes [59]:

- Initially, the usual dose of the basal insulin should be divided, and 2/3 of the dose should be administered before the morning and 1/3 of the dose before the evening meal.
- The insulin dose should be adjusted by adding 1 to 2 U or 10% to 15% once or twice a week until the target values in the glucose self-monitoring are obtained. In case of 4 blood glucose measurements per day, the insulin dose before the breakfast should be adjusted to control the blood glucose concentration after lunch and before supper, and the dose administered before the dinner should be changed to control the blood glucose measured before bedtime and before breakfast.
- If hypoglycemia occurs, the appropriate insulin dose should be reduced by 2 to 4 U or 10% to 20%.

Recommendations for choosing dosage of insulin mixtures are similar to those of basal insulin, and dose adjustments depend on the results of blood glucose measurements. It is assumed that patients treated with insulin mixtures should eat meals at a similar rate of calories. It should also be noted what part of the mixture is short-acting or fast-acting insulin. A randomized study performed by Chen et al. showed that insulin mixtures containing 50% of the fast-acting analogue are much more effective in treating patients using higher carbohydrate diets [62]. This is reflected in the summary of clinical recommendations on the use of insulin mixtures in the treatment of type 2 diabetes, indicating that in some patients Mix50 mixtures may be more appropriate than Mix25/30 mixtures, and clinicians should consider not only efficacy and safety, but also traits and patient preferences during insulin treatment of people with type 2 diabetes [63].

In healthy people after eating a meal, insulin releases quickly and significantly increases, which is accompanied by an increase in the secretion of amylin (another hormone besides insulin, which is produced by β cells), which inhibits the secretion of glucagon, slows gastric emptying and causes a feeling of satiety. An important role is also played by various gastro-intestinal peptides, including GLP-1, which also inhibit the release of glucagon, such as amylin, and slow the emptying of the stomach and lead to a feeling of fullness. In people with type 2 diabetes, the release of insulin after a meal is slower, no increase is observed as in healthy people, the peak of insulin secretion occurs 90 to 120 min after starting the meal whereas in healthy people it is observed within the first 30 min. The increase in amylin secretion is also delayed and reduced, which, as a consequence, does not lead to suppression of glucagon, slowing gastric emptying and limiting food intake (no feeling of fullness). The secretion or action of GLP-1 may also be impaired [64]. Many individuals with type 2 diabetes may require mealtime bolus insulin dosing in addition to basal insulin. The dose and duration of action of prandial insulin should correspond to the need for adequate glycemic control. In many cases, meals vary greatly in terms of composition, size and time of consumption. Inappropriate therapeutic decisions regarding insulin dosing may lead to significant hyperglycemia or hypoglycemia. It should also be remembered that the exogenous insulin injection leads to inhibition of endogenous insulin and amylin secretion, reducing the feeling of satiety, leading to increased food intake and the body weight gain. In the case of a significant reduction in endogenous insulin and amylin secretion, treatment with insulin administered before meals is very difficult.

A lack of ability to achieve adequate glycemic control (HbA1c) during the treatment with basal insulin is a signal, besides the possibility of treatment with insulin mixtures, for administration of a fast-acting analogue before the largest meal, i.e., the use of the basal-plus insulin regimen. Usually an analogue is administered at a dose of 4 U, 0.1 U/kg or 10% of the basal insulin dose. If HbA1c is lower than 8.0% (64 mmol/mol) during this period, a reduction in the basal insulin dose should be considered. Then the dose should be adjusted by 1 to 2 U or 10% to 15% once or twice a week until the target values in self- control are achieved. In the presence of hypoglycemia and the diagnosis of the cause, the dose is reduced by 2 to 4 U or by 10% to 20%. It is a simple treatment that is easily accepted by patients [59]. On average, after adding a single dose before one meal, a reduction of HbA1c by 0.3 to 0.5% (3.3 to 5.5 mmol/mol) is observed. At the beginning of the treatment, the addition of a single injection of insulin is usually just as effective as using two injections, but with a lower risk of hypoglycemia and similar glycemic control is achievable as in the case of insulin delivery in multiple injections [65,66]. A good therapeutic effect can be obtained regardless of the rate of change in adjusting insulin doses [67]. Hence, just like in the case of insulin mixtures, the dose setting depends on the results of glycemic measurements and it is not based on adjusting the doses to the meal. Along with the time from the diagnosis of type 2 diabetes, the postprandial hyperglycemia control becomes more and more difficult. Many patients require early significant intensification of therapy to ensure further decades of active life. In particular, earlier intensification of the treatment due to a rapid deterioration of metabolic control is required in relatively young people with type 2 diabetes [68]. However, intensification of insulin therapy increases the risk of weight gain and hypoglycemia. Many patients reach a state where further insulin dose increase does not improve glycemic control. In addition, as demonstrated in studies evaluating the results of intensification of insulin therapy in people with type 2 diabetes, the basal-bolus regimen provides a small further improvement in HbA1c levels compared to simpler insulin delivery regimens [69,70].

The ADA states in its recommendations that the postprandial glucose increase may be better controlled by adjusting the time of insulin administration before a meal, indicating that the type of insulin used (short acting insulin or a fast-acting analog) should be taken into account while adjusting the time of insulin administration. Insulin doses set before meals should depend on the measured blood glucose levels and meal times. In this case, ADA also draws attention to the adjustment of the dose according to the carbohydrates consumed. However, this is only a note, because in the published recommendations the results of glycemia have a major impact on the setting of insulin doses [59].

For over a dozen years, research has been undertaken to assess the effectiveness of insulin therapy in people with type 2 diabetes based on adjusting insulin doses to meals. In 2008, Bergenstal et al. published the results of a multicenter, controlled, open label, randomized study that compared the use of two algorithms, i.e., Simple Algorithm vs. Carbohydrates Count, for adjusting mealtime insulin along with a simple algorithm for adjusting glargine insulin in a group of people with type 2 diabetes. The study involved 273 participants aged 18–70 years, with type 2 diabetes for at least 6 months, with HbA1c in the range of 7.0 to 10.0% (53 to 86 mmol/mol), taking at least 2 insulin injections per day for at least 3 months before the study. All patients recorded results of the self-monitored blood glucose before meals and at bedtime, insulin doses, menus, information on hypoglycemia, physical activity levels as well as the results of a 7-point blood glucose profile performed on week 0, 12, 18, and 24. The study established fairly stringent blood glucose targets that were as follows: fasting < 5.3 mmol/L (95 mg/dL), preprandial (before lunch and dinner) < 5.6 mmol/L (100 mg/dL) and before sleep < 7.2 mmol/L (130 mg/dL). In the Carbohydrates Count group, depending on the sensitivity to insulin, from 1 U per 20 g of carbohydrates to 3 U per 15 g of carbohydrates were given. In this group, a slightly greater but not statistically significant reduction in HbA1c, i.e., −1.59 vs. −1.46% (−17.5 vs. −16.1 mmol/mol) was obtained, lower daily doses of insulin were used, and a tendency towards less weight gain was observed [71]. In AACE/ACE Consensus Statement it has been declared that “carbohydrate counting was not more effective than a simplified bolus insulin dosage algorithm based on pre-meal and bedtime glucose patterns” based on results of this trial [8]. However, Hirose et al. conducted tests in a hospital setting in a small group of patients comparing the results of dose setting based on glycemia and based on carbohydrate content counting. After 14 days of treatment, better glycemic control was obtained in the carbohydrate counting group (*p* < 0.001) [72]. These results were not confirmed by the authors of another randomized study, which compared the fixed meal insulin dosing with flexible meal dosing based on carbohydrate counting in hospitalized people with type 2 diabetes requiring at least 20 U of insulin per day. In the flexible meal dosing group, the algorithm used to treat type 1 diabetes with insulin pumps was adopted. The constant dose group required much larger amounts of basal insulin, but both groups achieved similar glycemic control [73]. During the last EASD meeting in Berlin (2018), a group of Danish researchers announced the results of the evaluation of efficacy of advanced carbohydrate counting and the use of an automated bolus calculator compared with mental insulin bolus calculation in people with type 2 diabetes on the basal-bolus insulin therapy. In conclusion they stated that the advanced carbohydrate counting and insulin bolus calculation is an efficient, low cost tool to reduce HbA1c in people with type 2 diabetes on the basal-bolus insulin regimen. Similar effects were observed regardless of whether the automated bolus calculator or the mental bolus calculations were used. The blinded continuous glucose monitoring revealed decreased glycemic variability with both options, whereas only the group using the automated bolus calculator increased the time in the euglycemic range [74].

Practically, the system based on the results of both glycemic and glycemic-after-prone tests is a combined treatment with GLP-1 agonist and the basal insulin. However, this treatment is effective only in those people with type 2 diabetes who have preserved endogenous insulin secretion.

## 6. Conclusions

It seems reasonable to conclude that in the insulin treatment of people with diabetes prandial doses of insulin should take into account the result of pre-meal glycemia as well as the composition and size of meals. There are still open questions regarding the optimal way to adjust a prandial insulin dose to a meal and the possible benefits for people with type 1 and type 2 diabetes if particular parameters of the meal are taken into account while calculating the prandial insulin dose (e.g., content of all macronutrients in a meal, the proportion and order of consumption of various foods during a meal, inter-subject variability of response to the meal in respect to the postprandial blood glucose etc.). It should be emphasized that the answers to these questions may vary depending on the type of diabetes. In type 1 diabetes, it is generally accepted and considered to be beneficial to take into account the carbohydrate content of the meals when adjusting the prandial insulin doses. There is also evidence that protein and fat counting is advantageous in children and adolescents with type 1 diabetes treated with insulin pumps. However, there is no data available on this topic for adults with type 1 diabetes or people with type 2 diabetes. Hence, we should emphasize that the following questions are still waiting for answers:

- Is the calculation of carbohydrate exchangers sufficient to achieve improved postprandial glycemic control in adults with type 1 diabetes and people with type 2 diabetes or should the intake of protein and fat be also taken into account?
- What other factors should be taken into consideration when determining doses of insulin administered before meals in people with diabetes?
- Is it possible to develop a simple algorithm for prandial insulin dose adjustment based on the blood glucose measurements and counting all macronutrients in the meal?
